# Pharmacokinetics of recombinant activated factor VII in trauma patients with severe bleeding

**DOI:** 10.1186/cc4977

**Published:** 2006-07-19

**Authors:** Thomas Klitgaard, Rene Tabanera y Palacios, Kenneth D Boffard, Philip TC Iau, Brian Warren, Sandro Rizoli, Rolf Rossaint, Yoram Kluger, Bruno Riou

**Affiliations:** 1 Department of Biomodelling, Novo Nordisk A/S, Maaloev, Denmark; 2 Department of Biostatistics, Novo Nordisk A/S, Bagsvaerd, Denmark; 3 Department of Surgery, Johannesburg Hospital, Houghton, Johannesburg, South Africa; 4 Department of Surgery, National University Hospital, Singapore; 5 Department of Surgery, Tygerberg Hospital, University of Stellenbosch, Tygerberg, South Africa; 6 Departments of Surgery and Critical Care Medicine, Sunnybrook and Women's College Health Sciences Centre, University of Toronto, Toronto, Ontario, Canada; 7 Department of Anesthesiology, University Clinics, Aachen, Germany; 8 Department of Surgery, Haifa Medical Center, Haifa, Israel; 9 Department of Emergency Medicine and Surgery and Department of Anesthesiology and Critical Care, Hôpital Pitié-Salpêtrière, Assistance Publique-Hôpitaux de Paris; Université Pierre et Marie Curie-Paris 6, Paris, France

## Abstract

**Introduction:**

Recombinant activated factor VII (rFVIIa) has been used as adjunctive therapy in trauma patients with severe bleeding. However, its pharmacokinetics profile remains unknown.

**Methods:**

In two placebo-controlled studies in patients with blunt and penetrating trauma, the pharmacokinetics of rFVIIa given at an initial dose of 200 μg.kg^-1 ^after transfusion of eight red blood cell units, followed by additional doses of 100 μg.kg^-1^, one and three hours later, have been studied, based on the FVII coagulant activity assay. Both non-compartment and population pharmacokinetic analyses were performed. A two-compartment, population pharmacokinetic model was used to estimate a population profile for the rFVIIa dosing regimen. Data are population means (percent coefficient of variation (CV)).

**Results:**

Based on the two-compartment population model, the estimated pharmacokinetic parameters were: clearance 40 (30% CV) ml.kg^-1^.h^-1^; central volume of distribution 89 (32% CV) ml.kg^-1^; inter-compartmental clearance 24 ml.kg^-1^.h^-1^; and peripheral compartment volume 31 ml.kg^-1^. Baseline FVII coagulant activity was estimated at 0.29 (39% CV) U.ml^-1^, initial half-life was 0.6 (34% CV) hours, and terminal half-life 2.4 (50% CV) hours. High intra- and inter-patient variability was noted in volume of distribution and clearance, which was in part correlated with the transfusion requirements as the single significant covariate. The non-compartmental analysis led to almost identical estimates of key parameters.

**Conclusion:**

A high intra- and inter-patient variability was noted in the volume of distribution and clearance of rFVIIa in trauma patients with severe bleeding, mainly related with the transfusion requirements and thus blood loss and/or bleeding rate.

## Introduction

Recombinant activated factor VII (rFVIIa, NovoSeven^®^/Niastase^®^, Novo Nordisk A/S, Bagsvaerd, Denmark) is an effective first-line hemostatic agent for the management of acute and surgical bleeds in patients with hemophilia A or B and inhibitors to factor VIII or factor IX [1]. The ability of rFVIIa to provide effective hemostasis in patients with a variety of clinical conditions associated with bleeding can be attributed to its capacity to increase thrombin generation on the surface of activated platelets accumulated at the site of injury to the vessel wall, and, by directly activating factor X in the absence of tissue factor, this agent promotes the formation of a tight, stable fibrin plug at the site of injury [2,3]. An expanding literature suggests that rFVIIa has the potential for broad-spectrum applications in situations characterized by profuse bleeding and impaired thrombin generation [4-6], including severe trauma [7,8], although some other studies suggest that its preventive use in potentially bleeding situations may not be appropriate [9].

A randomized study in trauma patients recently demonstrated that adjunctive therapy with rFVIIa controls bleeding, resulting in a significant reduction in red blood cell (RBC) transfusion requirements and in the occurrence of massive transfusion [10]. In the trauma population it is difficult to measure the amount of blood loss accurately. Accordingly, units of RBC transfused was chosen as a surrogate endpoint for bleeding, and units of RBC transfused during the 48 hour observation period following the initial dose of trial product was chosen as the primary endpoint to indicate the ability of rFVIIa to control bleeding [10]. Among the patients with blunt trauma and surviving the initial 48 hours after first dose, RBC transfusion was significantly reduced with rFVIIa relative to placebo (estimated reduction of 2.6 RBC units, *P *= 0.02), and the need for massive transfusion (>20 RBC), a *post hoc *analysis, was also significantly reduced (14% versus 33% of patients, *P *= 0.03). In penetrating trauma, similar analyses showed trends towards rFVIIa reducing RBC transfusion (estimated reduction of 1.0 RBC units, *P *= 0.10), and massive transfusion (7% versus 19% of patients, *P *= 0.08) [10].

Clinical studies have not identified pharmacodynamic markers that can reliably predict the *in vivo *hemostatic effect of rFVIIa. Instead, dose selection is guided by *in vitro *studies with human biomaterials, clinical experience, among other from studies in patients with hemophilia [3,11-14], and an understanding of the time course of the drug levels with dose, that is, the pharmacokinetics.

The objectives of our study were to address the latter issue, using two different analytical approaches to describe the pharmacokinetic profile of rFVIIa in trauma patients with severe bleeding. Moreover, our aim was also to identify significant and clinically relevant covariates affecting pharmacokinetics in this population. The present study is an ancillary pharmacokinetic study of the large clinical trial that has been recently published [10].

## Materials and methods

The study protocol was approved by the ethics committee of each participating institution, and the trial was conducted according to Good Clinical Practice standards and the Helsinki Declaration. Written, informed consent was obtained from all patients, or, where applicable, from a legally authorized representative. Due to the emergency conditions and the possible absence of relatives at enrolment into the trial, waived informed consent was authorized by the ethical committees. However, whenever a patient was included without written informed consent, such consent was promptly searched from a legally authorized representative and subsequently from the patient himself. When adequate confirmation of consent was not obtained, data were excluded from analysis.

The methods of the study have been previously detailed [10]. Briefly, to be eligible for inclusion, patients were to have received six units of RBC within a four-hour period, and to be of known age = 16 years (or legally of age according to local law) and <65 years. Key exclusion criteria comprised: cardiac arrest pre-hospital or in the emergency or operating room prior to trial drug administration; gunshot wound to the head; Glasgow coma scale <8 unless in the presence of a normal head CT scan; base deficit of >15 mEq.l^-1 ^or severe acidosis with pH <7.00; transfusion of eight units or more of RBC prior to arrival at trauma center; and injury sustained = 12 hours before randomization.

This was a randomized, placebo-controlled, double-blind trial with two parallel treatment arms in two separate trauma populations. Patients were evaluated for inclusion into the trial on admission to the trauma center, and eligible patients were assigned to either the blunt or penetrating trauma trial arm. Upon receiving six units of RBC within a four-hour period, eligible patients within each trauma population were equally randomized to receive either three intravenous injections of rFVIIa (200, 100 and 100 μg.kg^-1^) or three placebo injections. The first dose of trial product was to be administered immediately after transfusion of the eighth unit of RBC, given that the patient, in the opinion of the attending physician, would require additional transfusions. The second and third doses followed one and three hours after the first dose, respectively. This dose regimen was chosen to target an average concentration of FVIIa >40 U.ml^-1^, based on *in vitro *studies and clinical studies conducted in hemophilic patients. Trial product was administered in addition to standard treatment for injuries and bleeding at the participating hospitals, and no restrictions were imposed on procedures deemed necessary by the attending physician, including surgical interventions, resuscitation strategies, and use of blood products. However, before patient enrolment, each participating trauma center developed specific transfusion guidelines in line with the transfusion guidelines provided in the study protocol.

### FVII coagulant activity assay

All pharmacokinetic assessments in this study were based on the FVII coagulant activity assay, a one-stage clotting assay using thromboplastin tissue factor, which forms complexes with both FVIIa and FVII zymogen to quantify FVII clotting activity in plasma (Capio Diagnostik A/S, Copenhagen, Denmark) [15]. The lower limit of quantification for the assay is 0.06 U.ml^-1^. The assay involves use of diluted samples of plasma that were then mixed with FVII-deficient plasma. Temperature was then adjusted to 37°C, and coagulation was initiated by the addition of thromboplastin tissue factor and calcium chloride. The time until fibrin formation was measured and related to the time observed for this reaction in normal plasma. Due to the temperature adjustment and the sample dilution in buffer and FVII-deficient plasma (which has a high buffer capacity), no effect of hypothermia or acidosis on assay results are expected. Since the FVII coagulant activity assay does not distinguish endogenous FVII/FVIIa from rFVIIa, baseline plasma activity (that is to say, before administration of rFVIIa) was taken into account in the pharmacokinetic analyses. The therapeutic doses of rFVIIa used in the study were expected to give peak plasma concentrations at least 30-fold greater than the normal endogenous FVII/FVIIa level in non-coagulopathic patients.

### Pharmacokinetic sampling

Within the two study populations of blunt and penetrating trauma, subjects were allocated to two groups for pharmacokinetic analysis, one group to frequent blood sampling and another to sparse blood sampling for determination of FVII coagulant activity assay. The frequent sampling group comprised approximately 50 patients from whom plasma was sampled before first dose of trial product and 30 minutes and 1, 2, 3, 4, 6, 8 and 12 hours after the first dose. The remaining patients formed the sparse sampling group, from whom one sample was taken in each of at least two of the following four time intervals: 0 to 1 hour (after the first dose but before the second dose); 1 to 3 hours (after the second dose but before the third dose); 3 to 8 hours (after the third dose); and 8 to 12 hours.

### Pharmacokinetic analyses

Data from patients with frequent sampling were analyzed non-compartmentally, whereas data from both patients with frequent and patients with sparse sampling were used for population pharmacokinetic analysis. The latter approach – population analysis – is suitable for this type of data. In contrast to the non-compartmental analysis – which is based upon separate analysis of each individual profile and requires that enough data be available for each individual to actually estimate the individual pharmacokinetics profiles – the population approach does not have this constraint. Instead, even very sparsely sampled individual profiles can be included in the dataset, in addition to the more richly sampled profiles. Eventually, an overall 'population pharmacokinetic profile' is estimated, as are estimates of how the parameters describing this profile vary between individuals. Using this approach, the effect of a number of covariates on individual parameters may also be analyzed [16,17].

As mentioned, the FVIIa coagulant assay does not distinguish endogenous FVII/FVIIa from rFVIIa. Although very small in comparison with the levels obtained during FVIIa treatment, a baseline FVIIa coagulant activity level was estimated for each individual and adjusted for in the analysis. In the non-compartmental analysis, the level was estimated based on pre-dose FVIIa coagulant activity levels and subtracted from the post-dose measurements; in the population analysis, the level was included as a subject specific random effect and estimated as part of the model estimation procedure. The mean baseline level was estimated at 0.30 and 0.29 U.ml^-1 ^for the placebo and the treatment group, respectively (non-compartmental estimates).

#### Non-compartmental analysis

The following parameters were estimated: maximum plasma FVII coagulant activity from time of first dose (time zero) to 12 hours after first dose (C_max_); time to maximum plasma FVII coagulant activity (T_max_); area under the plasma FVII coagulant activity-time profile from time of first dose (time zero) to 12 hours after first dose (AUC_0–12h_); and volume of distribution and clearance. For calculation of AUC_0–12h_, adjusted activities below zero were substituted by zero.

#### Population analysis

In this analysis, both a one- and a two-compartment population model were explored. Since the latter type was found to better describe the data, the final model was a two-compartment population model with first order elimination from the central compartment and baseline FVII coagulant activity assay to account for endogenous production of FVIIa. The influence of the covariates RBC transfusion, body weight, type of injury (blunt or penetrating), injury severity score, sex, age, and ethnicity on the model parameters were tested during development of the model. These covariates, based on the available data, were *a priori *identified as clinically relevant in collaboration with the medical team. Results were compared to population analysis results obtained in a previous study in hemophilia patients (Novo Nordisk data on file). In this analysis, pharmacokinetic data previously analyzed non-compartmentally [18] were analyzed with a two-compartment population pharmacokinetic model similar to the one presented here to allow for simulation of the population pharmacokinetic profile in hemophilia patients.

The final population pharmacokinetic model was used to simulate the predicted mean profile for the trauma population, at different levels of post-dose RBC transfusion requirements, as clearance was found to correlate with this covariate.

### Statistical analysis

Data are expressed as geometric and population means (non-compartmental and population analysis, respectively), percent coefficient of variation (%CV = standard deviation × 100/mean) for pharmacokinetic parameters and arithmetic mean ± standard deviation for other variables. All statistical comparisons were based on analysis of variance methods, using two-tailed tests and a significance level of 0.05.

For the non-compartmental analysis, data file preparation and statistical analysis was performed with SAS version 8.2 (SAS Institute Inc., Cary, NC, USA). For the population pharmacokinetic analysis, data file preparation was performed with SAS Version 8.2 and S-PLUS v. 6.0 for Trial Simulator (Insightful Corporation, Seattle, WA, USA); non-linear mixed effects modeling was performed with NONMEM version 5.11 (GoboMax, Hanover, MD, USA), run under Visual-NM Version 5.1 (RDPP, Montpellier, France). Visual Fortran Version 6.1 (Hewlet-Packard Company, Palo Alto, CA, USA) was used for compiling and clinical trial simulation was performed with the Pharsight Trial Simulator v. 2.1.2 (Pharsight Corporation, Mountain View, CA, USA). Software was installed according to vendor instructions. In addition, NONMEM functionality was verified with a test script executed before modeling.

NONMEM's first order conditional estimation method with interaction was used for model development. Evaluation and discrimination of intermediate models was based on standard statistical theory that the change in objective function value is approximately Chi-square distributed, with degrees of freedom corresponding to the difference in number of parameters [19]. Covariates were included in a forward inclusion approach, if reduction in objective function value was significant at a *P *value of 0.05. Subsequently, covariates were excluded by backwards elimination from the full model, if the associated increase in objective function value was not significant at a *P *value of 0.001. This method ensured inclusion of the relevant covariates, and the latter highly conservative significance level was employed to retain only the most essential covariates, leading to a more robust model.

Between and within patient variability was estimated as part of the model estimation procedure in NONMEM. The between-patient variation of parameter estimates was based upon the assumption of individual parameter estimates being log-normal distributed around a population mean and is based on the inclusion of a random subject effect for the parameter. The number of parameters in the model must be balanced with the amount of data. Thus, to avoid over-parametrization of the final model, a random effect was not included for the inter-compartmental clearance and peripheral compartment volume. For these parameters, between patient variability is hence not reported. Within patient variation was estimated from the log normal distribution of individual observations around the individually predicted pharmacokinetic profiles. Both were expressed as %CV. Confidence intervals for the parameter estimates were calculated on the log-scale, as estimates ± 1.96 standard deviation.

## Results

A total of 301 patients were randomized in the two studies, 158 in the blunt trauma arm and 143 in the penetrating trauma arm. Of these, 18 were withdrawn before administration of trial product, and waived informed consent was not confirmed for 6 patients. Of the remaining 277 patients, the pharmacokinetic profiles from a total of 47 patients were excluded from the dataset for the following reasons: no recording of actual sampling time (*n *= 15); no FVII coagulant activity recordings (*n *= 13); apparently artifactual pre-dose FVII coagulant activity levels or other aberrant data values (*n *= 3); and lack of information on potentially relevant covariates in the population model (*n *= 16). There were no significant differences between excluded and included patients in age, sex, Injury Severity Score, RBC requirement and survival (data not shown), suggesting that our analysis was not affected by patient exclusion bias. In the final pharmacokinetic dataset of 230 patients, 107 had been treated with placebo while the remaining 123 patients had been treated with rFVIIa. The population was characterized by the following characteristics: mean age was 32 ± 11 years; mean Injury Severity Score was 28 ± 13; there were 191 (83%) men and 39 (17%) women; and there were 110 (48%) patients with blunt trauma and 120 (52%) patients with penetrating trauma.

Before performing the actual pharmacokinetic analyses, the baseline level of FVIIa activity was explored, by plotting the activity versus time for the 107 subjects treated with placebo. The level appeared to be relatively constant in time, with random fluctuations around an average level of 0.3 U.ml-1 (range, 0.07 to 1.4 U.ml-1). Based on this, baseline levels were assumed individual specific, but constant in time.

### Non-compartmental analysis

**Figure 1:**
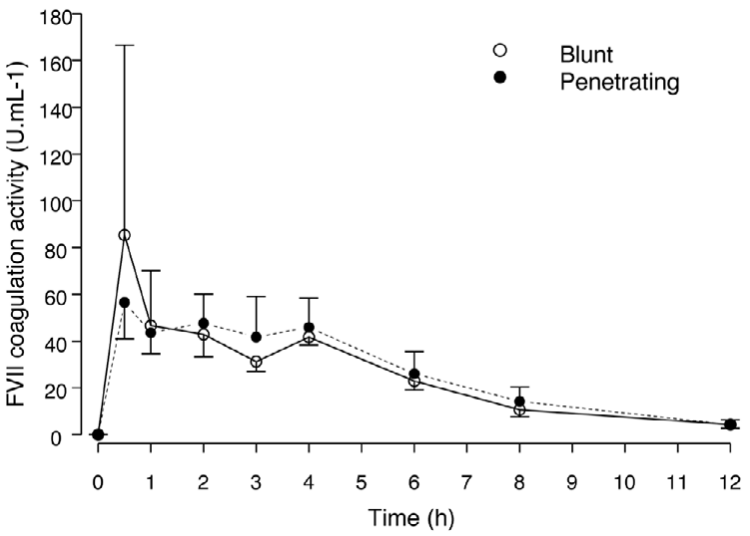
Factor VII (FVII) coagulant activity measurements in recombinant FVIIa-treated blunt (*n *= 6) and penetrating (*n *= 15) trauma patients. One outlier was excluded from the population pharmacokinetic model data set.

**Figure 2:**
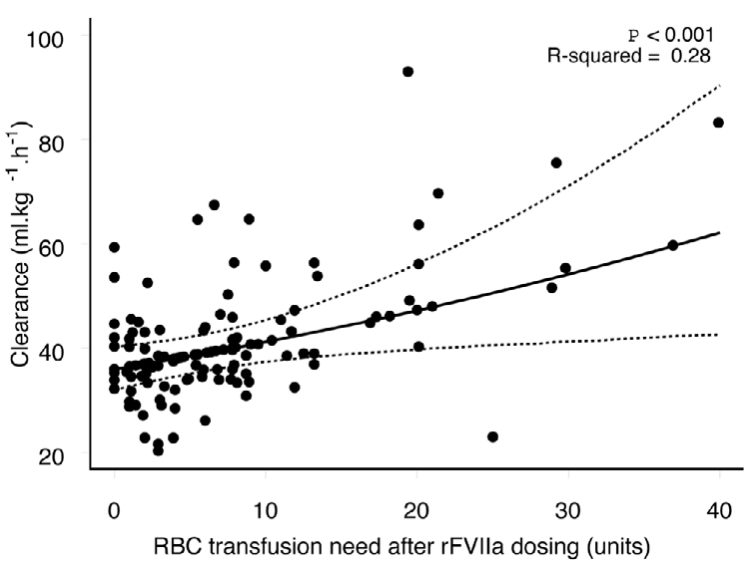
Correlation between clearance and red blood cell (RBC) transfusion requirement after first dose of recombinant factor VIIa (rFVIIa). Plot of model-estimated, individual clearance versus measured post-dose RBC requirement (*n*= 123). R-squared value reflects the fraction of the variation in individual predicted clearance values explained by the model. *P *value reflects the significance level at which the hypothesis of no effect of RBC transfusion need after rFVIIa dosing on individual clearance was rejected. Dotted lines indicate 95% confidence interval of the regression curve.

The frequent sampling group, for which the non-compartmental pharmacokinetic analysis was performed, comprised 43 patients, of which 18 patients were treated with placebo and 25 patients were treated with rFVIIa. Analysis was performed only on rFVIIa-treated patients with at least five plasma FVII coagulant activity measurements yielding data from six blunt and 15 penetrating trauma patients (Figure 1). Results of the non-compartmental analysis are summarized in Table 1. No significant differences were noted in key pharmacokinetic parameters between patients with blunt and penetrating trauma. The uncertainty of the estimates of C_max_, T_max_, and volume of distribution was rather high, in particular for the group of patients with blunt trauma, which was not unexpected, since only a few patients contributed to these. Mean FVII coagulant activity (blunt and penetrating groups) are shown in Figure 2.

### Population analysis

In this analysis, data from 230 patients with frequent sampling and sparse sampling were used, of whom 123 were treated with rFVIIa. Diagnostics plots for the final population pharmacokinetic model indicated an acceptable fit of the model to the data, considering the amount of and variation in data (data not shown). The parameter estimates for the final population pharmacokinetics model are presented in Table 2. Population mean parameters for central compartment volume of distribution, clearance, and baseline are estimated with good precision and with estimates of the between-patient variation. Population mean parameters for inter-compartmental clearance and peripheral compartment volume are estimated with somewhat lower precision. For this latter set of parameters, the data did not allow for the estimation of inter-individual variation. Residual intra-individual variation was estimated at 32%.

For clearance, a significant part of the variation (*P *< 0.001) was attributable to differences in RBC transfusion requirements of the patients (Figure 2). Consequently, this clinically relevant correlation was included in the final model by the equation:

CL = 40.45 × 1.014^(RBC-8.7)^

where CL is the clearance, RBC is the RBC requirement after the first dose of rFVIIa and 8.7 is the average post-dose RBC requirement of the trauma population, indicating that the clearance increases with increasing RBC transfusion requirement.

### Application of the model

**Figure 3:**
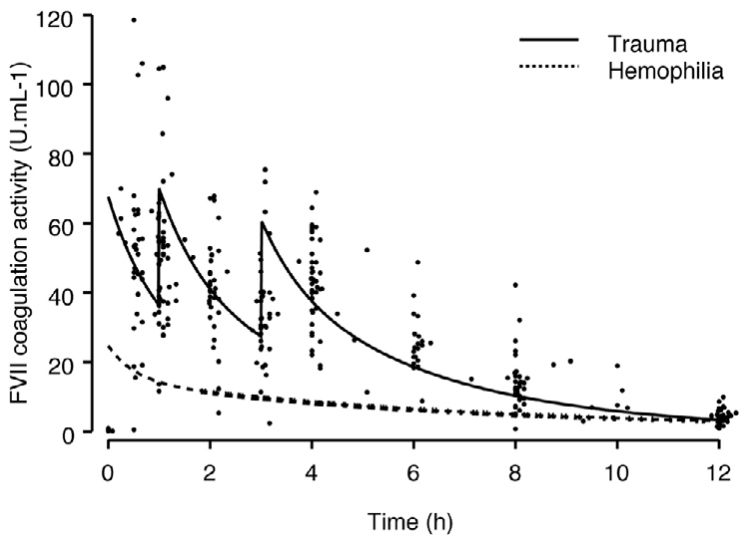
Population factor VII (FVII) coagulant activity profile modeled from the study dosing regimen. Dots represent the observed FVII coagulant activities from both frequent and sparse sampling while the solid line is trauma average population profile for multiple dosing. This model shows dosing in an adult hemophilia population superimposed for comparison (see Materials and methods).

The final model was used to simulate the population pharmacokinetics profile (Figure 3). The observed data were found to be quite variable. Nevertheless, most patients, based on data from the trauma population and the estimated population pharmacokinetics profile, achieved FVII coagulant activity at least equal to or above the pharmacokinetics profile reached in hemophilia patient populations given a single dose of 90 μg.kg^-1^. Within the first four hours after the first dose, only a few patients (*n *= 10, 12%) had plasma concentrations below the hemophilia profile (Figure 3), and 30 (75%), 40 (63%), and 32 (54%) patients achieved rFVIIa plasma concentrations above 40 U.ml^-1 ^after the first, second, and third boluses, respectively.

**Figure 4:**
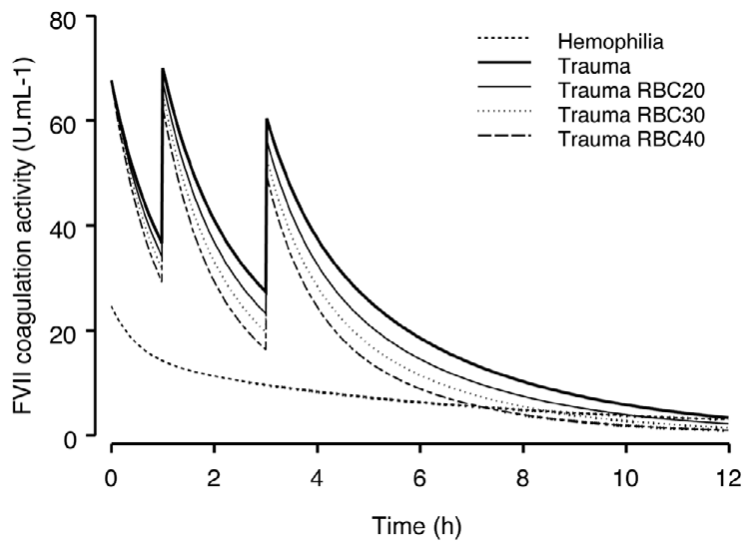
Population pharmacokinetics profiles simulated at various red blood cell (RBC) transfusion requirements (20, 30, and 40 units after dosing) – increasing transfusion requirement linked with increasing clearance. The full line depicts the global trauma population (Trauma, mean of 8.7 RBC units) Data for a hemophilia population has been superimposed for comparison(see Materials and methods).

As mentioned above, RBC transfusion requirement was the single significant covariate in the model. To illustrate the impact of this covariate, estimates of population clearance and terminal half-life at various post-dose RBC requirements were calculated. Increased RBC requirements were associated with increased clearance, and consequently with shortening of the terminal elimination half-life (Table 3). For comparison, estimates of clearance and half-life for patients with hemophilia are also presented. To further explore the effect of the covariate, population profiles were simulated for various levels of post-dose RBC transfusion requirements (Figure 4). Consistent with the observed data, the results indicated that for the average trauma population with a post-dose requirement of 8.7 units of RBC (population average), peak plasma levels of rFVIIa activity equivalent to approximately 65 U.ml^-1 ^(equal to approximately 43 nM) may be expected. Moreover, the average level appeared to remain above 40 U.ml^-1 ^(approximately 26 nM) for most of the four hours after the initial dose. In comparison with this, for subjects with an estimated post-dose RBC transfusion requirement of 40 units, the predicted level of coagulant activity displayed a significantly faster decline and approached, but did not fall below, the profile for the hemophilia population (Figure 4).

The variation in FVIIa coagulant activity in the trauma population was considerable. Part of this variation was attributable to differences in RBC transfusion requirement, resulting in significantly different predicted pharmacokinetic profiles depending on this covariate. As reflected in the data (Figure 3) and the population pharmacokinetic model parameters (Table 2), the remaining variation not accounted for was still considerable; however, with the dosing schedule used in this study, it appears that even trauma subjects with high distribution volumes and clearance (compared with population average) will achieve plasma levels of rFVIIa activity that do not fall below levels seen in the hemophilia populations managed with a single dose of 90 μg.kg ^-1^.

## Discussion

In this large prospective study evaluating the pharmacokinetic properties of rFVIIa in trauma patients with severe bleeding, we mainly observed that: the mean clearance was 40 ml.kg^-1^.h^-1 ^and the terminal half-life 2.4 hours; a high intra- and inter-patient variability was noted in the volume of distribution and clearance; and this high variability was significantly correlated with the transfusion requirements and thus blood loss. The pharmacokinetic analyses reported here complete our previous report on the clinical efficacy and safety data, which suggested that, in severe blunt trauma patients, a dosing schedule for rFVIIa of 200 μg.kg^-1 ^followed one and three hours later by additional doses of 100 μg.kg^-1 ^in patients with severe bleeding is an effective hemostatic therapy [10].

Although definite conclusions could not be drawn from the non-compartmental pharmacokinetic analysis due to the small number of patients analyzed, profiles derived using this method of analysis were valuable in giving an essentially model-free interpretation to the population data. The observed variability in the results of C_max _was expected, due the variation in distribution volume and clearance. The mean clearance of approximately 40 ml.kg^-1^.h^-1 ^noted in the non-compartmental analysis was almost identical to that seen in the population pharmacokinetics analysis. Pharmacokinetic population modeling and analysis was successful in describing the profile of rFVIIa pharmacokinetics in trauma patients, in terms of a two-compartment model. For a few parameters in the model (intercompartmental clearance and peripheral compartment volume), inter-subjects variation was not estimated. Moreover, the precision of the corresponding population mean parameter estimates was relatively low, compared with the other parameters in the model. However, considering the amount and quality of the data available, this was not unexpected and was considered satisfactory.

The estimated population pharmacokinetics profile at a post-dose RBC requirement of 8.7 units (the population average) indicated that, after three doses of rFVIIa, the peak plasma FVII coagulant activity of the population pharmacokinetics profile was approximately 65 U.ml^-1 ^(43 nM). Furthermore, the plasma level appeared to remain above 40 U.ml^-1 ^(approximately 26 nM) for most of the time over the four hours after the initial dose (Figure 3). The pharmacokinetic modeling and analysis as described here highlight that the trauma population constitute a group of patients with very high intra- and inter-patient variation in terms of rFVIIa kinetics. This may well reflect more than just differences in RBC transfusion requirements and underline the difficulties in attempting to treat such a diverse group of patients using a one-regimen-for-all approach to treatment. The population pharmacokinetic analysis illustrates the variation between trauma patients in terms of the rFVIIa pharmacokinetics, a variation that, in turn, has an effect on which dose will be required for obtaining and maintaining an effective FVII coagulant activity. Our analyses suggest that the chosen dose regimen will yield adequate plasma FVII coagulant activity during the crucial treatment period even when volume of distribution and plasma clearance are elevated.

The population model describing the study population as a whole was appropriate for individual patients and helped identify covariates that could explain some of the pharmacokinetic variability noted in this trauma population. In spite of this high variation, a significant correlation was found, in that clearance of rFVIIa was found to increase with increasing RBC requirement (Figure 2), most likely due to bleeding and plasma volume replacement. In theory, if a trauma patient had no post-dose RBC requirement, the estimated clearance of rFVIIa would be 36 ml.kg^-1^.h^-1 ^and the terminal half-life would be 2.6 hours. These estimates are in good agreement with non-compartment and population analysis results reported in (non-bleeding) healthy volunteers [20,21]. However with an RBC requirement of 40 units, clearance almost doubles to 62 ml.kg^-1^.h^-1 ^while the half life is shortened by nearly one hour to 1.7 hours. When population pharmacokinetic profiles were simulated at various post-dose RBC requirements, it was found that an increase in RBC requirement correlated with a more rapid decrease in the predicted FVII coagulant activity. Furthermore, predicted peak plasma activities after second and third doses of rFVIIa were reduced as RBC requirements increased, and overall exposure to rFVIIa – as assessed by area under the FVII coagulant activity-time profile – was also reduced. These results are not surprising since hemorrhage is well known to markedly modify pharmacokinetic parameters [22,23]. Although not based on baseline information – since the RBC is measured after the first dose of rFVIIa – this correlation reflects a clinically relevant interplay between the measured FVII coagulant activity and the RBC transfusion volume and may help clinicians in choosing an appropriate dose according to the clinically estimated blood loss and/or bleeding rate. Therefore, our pharmacokinetic model may help to target appropriate rFVIIa concentrations in future randomized trials in other clinical conditions, such as postoperative bleeding [24,25]. Based on simulations using various post-dose RBC transfusion requirements and the observed individual levels of FVIIa coagulant activity in the study, it can, however, be anticipated that with the dosing schedule employed in this study, even patients with high distribution volumes and/or high RBC transfusion requirements due to severe bleeding will achieve a FVII coagulant activity at least equal to that known to be clinically effective in hemophilia settings.

Clinical studies with rFVIIa have not identified pharmacodynamic markers that reliably predict the *in vivo *hemostatic effect of rFVIIa; thus, measures such as prothrombin time and activated partial thromboplastin time are poor indicators of bleeding control in the coagulopathic patient [8]. It is important, therefore, to establish, from existing clinical data derived from trauma cohorts, that the total dose and dosing schedules for rFVIIa as evaluated in controlled studies are effective in achieving a pharmacokinetic profile of plasma FVII coagulant activity that supports the observed clinical efficacy of this hemostatic agent in trauma patients. The analysis presented here reveals some important aspects of the pharmacokinetics in this population, in terms of how variable it is and which factors may help in explaining some of this variation. But it also demonstrates that the dosing regimen chosen leads to FVIIa coagulant activity levels known to be efficient in the hemophilia population, for most subjects in the population, even those with above-average volumes of distribution and plasma clearance. This result is important since considerable variation in the range of doses of rFVIIa (from 40 to 300 μg.kg^-1^) has been noted in previously reported case series in trauma [7,8,26,27].

Some limitations in our study deserve consideration. First, only one dosing schedule for rFVIIa was studied and the question remains of whether the same level of efficacy could be achieved with lower total doses or a different regimen of rFVIIa dosing [10]. The pharmacokinetic analysis described here is obviously limited by this fact; but it does demonstrate that a satisfactorily high level of FVII coagulant activity is obtained with the chosen regimen, compared with the levels seen in the hemophilia population, when managed with 90 μg/kg. Therefore, based on the results of the pharmacokinetic analysis described in this study, and the safety and efficacy outcomes as described by Boffard and colleagues [10], a phase III multi-center randomized placebo-controlled clinical trial investigating rFVIIa in severely injured trauma patients with bleeding refractory to standard treatment is currently ongoing with exactly the same dosing regimen as described here (that is to say, 200 + 100 + 100 μg.kg^-1 ^at hours 0, 1, and 3, respectively), in order to achieve appropriate levels of rFVIIa in the study population.

Second, there are no clear data concerning the optimal duration of adequate rFVIIa concentrations required in bleeding trauma patients. This concept is difficult to test in the context of a multi-center randomized placebo-controlled clinical trial, as the clinician decision that 'bleeding has stopped' is likely to be highly subjective. In the case series of 81 patients described by Dutton and colleagues [8], among the 46 subjects with acute hemorrhagic coagulopathy, doses of rFVIIa employed ranged from 48 to 148 μg.kg^-1 ^and patients in the series received an average of 1.2 (range 1 to 3) doses of rFVIIa. However, it should also be emphasized that, in this series, 25% of patients did not adequately respond to rFVIIa administration [8]. Therefore, the need for re-injection in selected patients remains a matter of debate in trauma patients and may ultimately depend on whether bleeding control has been achieved in a given patient.

Third, the average concentration targeted in our study (>40 U.mL^-1^) was based on *in vitro *studies and clinical studies conducted in hemophilic patients. Further evidence is required to confirm this average target concentration in severely bleeding patients. Nevertheless, it is notable that the only available randomized study that proved the efficacy of rFVIIa in trauma patients targeted this concentration [10].

Fourth, a larger amount of data would have enabled a more precise estimation of the model parameters and possibly also inclusion of intra-subject variance on all primary parameters. Lastly, pharmacokinetic models usually assume the volume and rate constants remain fixed for the duration of the experiments and, in our study, we modeled rFVIIa in a non-stationary system. We have tried to account for this by including RBC transfusion requirement as a covariate. However, the variability inherent in this clinical setting probably still adds to the intra- and inter-patient variabilities [28].

## Conclusion

In trauma patients with severe bleeding, the mean clearance of rFVIIa was 40 ml.kg^-1^.h^-1 ^and its terminal half-life 2.4 hours. A high intra- and inter-patient variability was observed in the volume of distribution and clearance of rFVIIa, mainly related with the transfusion requirements. This pharmacokinetic analysis completes our previous report on the clinical efficacy and safety of rFVIIa in trauma patients with severe bleeding [10] and may help to determine the precise, appropriate dosing regimen in future trials and clinical practice. Our study suggests that dosing might be adapted to the clinically estimated blood loss and/or bleeding rate.

## Key messages

• 	In trauma patients with severe bleeding, the mean clearance of recombinant factor VIIa is 40 ml.kg^-1^.h^-1 ^and its terminal half-life 2.4 hours.

• 	A high intra- and inter-patient variability occurs in the volume of distribution and clearance of recombinant factor FVIIa in trauma patients with severe bleeding.

• 	This high variability was mainly related to the transfusion requirements and thus the blood loss and/or bleeding rate

• 	Our study suggests that dosing might be adapted to the clinically estimated blood loss and/or bleeding rate in future trials and clinical practice.

## Abbreviations

AUC = area under the curve; C_max _= maximum concentration; CV = coefficient of variation; RBC = red blood cell; rFVIIa = recombinant factor VIIa; T_max _= time to maximum concentration.

## Competing interests

KB, SR, YK, and BR have received consultancy fees and lecture sponsorships from Novo Nordisk. RR has received lecture sponsorship from Novo Nordisk. TK and RTyP are employed by Novo Nordisk A/S.

## Authors' contributions

TK and RTyP designed and performed the pharmacokinetic analyses. TK, RTyP, and BR drafted the manuscript. All authors participated in the design and coordination of the study, and read and approved the final manuscript.

## Figures and Tables

**Table 1:** Pharmacokinetic parameters of factor VII coagulant activity assessed by non-compartmental analysis in blunt and penetrating trauma patients with frequent sampling

	Blunt (*n *= 6)	Penetrating (*n *= 15)
C_max _(U.ml^-1^)	81 (47–241) [76%]	58 (41–102) [27%]
T_max _(h)	0.9 (0.5–4.0) [110%]	1.2 (0.5–4.0) [86%]
AUC_0–12h _(U.h.ml^-1^)	305 (216–362) [17%]	311 [182–520] [26%]
Clearance (ml.kg^-1^.h^-1^)	41 (34–58) [21%]	40 (25–79) [30%]
Volume of distribution (ml.kg^-1^)	83 (28–143) [45%]	112 (66–165) [24%]

Data are geometric means (min-max) and coefficient of variation [%]. There is no significant difference between the two groups. AUC_0–12h_, area under the plasma concentration-time profile from time of first dose (time zero) to 12 hours after first dose; C_max_, maximum plasma concentration; T_max_, time to maximum plasma concentration.

**Table 2:** Population pharmacokinetic model parameter estimates (*n *= 230)

Variable	Estimate (95% CI)	Between patient CV
Clearance (ml.kg^-1^.h^-1^)	40 (37–44)	30%
Central compartment volume (ml.kg^-1^)	89 (60–132)	32%
Inter-compartmental clearance (ml.kg^-1^.h^-1^)	24 (8–76)	NA
Peripheral compartment volume (ml.kg^-1^)	31 (13–74)	NA
Baseline concentration (U.ml^-1^)	0.29 (0.27–0.31)	39%
ΔClearance/RBC	1.014 (1.003–1.0025)	NA
Initial half-life (h)	0.6 (0.2–1.3)	NA
Terminal half-life (h)	2.4 (1.8–5.5)	NA

The within-patient variability (random error) was estimated to be 32%. ΔClearance/RBC is the change in clearance per unit of red blood cells based on the model-specified potency function. CI, confidence interval; CV, coefficient of variation; NA, not applicable.

**Table 3:** Estimates of population average clearance and terminal half-life at various post-dose red blood cell requirements

Population	RBC requirements (Units)	Estimated clearance (ml.kg^-1^.h^-1^)	Terminal half-life (h)
Hemophilia^a^	NA	29	4.4
Trauma	0	36	2.6
	8.7^b^	40	2.4
	20	47	2.1
	30	54	1.9
	40	62	1.7

^a^Obtained in patients with hemophilia (see Materials and methods). ^b^This value corresponds to the trauma population average. NA, not applicable; RBC, red blood cell.
